# Computed Tomography (CT) Calcium Scoring in Primary Prevention of Acute Coronary Syndrome and Future Cardiac Events in Patients With Systemic Lupus Erythematosus

**DOI:** 10.7759/cureus.47157

**Published:** 2023-10-16

**Authors:** Michael Wu, Sophia Mirkin, Stephanie Nagy, Marissa N McPhail, Michelle Demory Beckler, Marc M Kesselman

**Affiliations:** 1 Osteopathic Medicine, Nova Southeastern University Dr. Kiran C. Patel College of Osteopathic Medicine, Fort Lauderdale, USA; 2 Rheumatology, Nova Southeastern University Dr. Kiran C. Patel College of Osteopathic Medicine, Fort Lauderdale, USA; 3 Microbiology and Immunology, Nova Southeastern University Dr. Kiran C. Patel College of Allopathic Medicine, Fort Lauderdale, USA

**Keywords:** acute coronary syndrome, atherosclerosis, cytokines, coronary artery calcium scoring, systemic lupus erythromatosus

## Abstract

Systemic lupus erythematosus (SLE) is a complex and chronic autoimmune disease that impacts multiple organ systems and presents with varying symptomatology that makes targeting treatment extremely difficult. The cardiovascular system and more specifically the coronary arteries are heavily affected by SLE causing increased atherosclerosis and subsequently increased acute coronary syndrome (ACS) and increased future cardiac events. ACS is a common occurrence in patients with SLE due to the premature development of atherosclerosis due to the dysregulation of pro-inflammatory cytokines. Calcium scoring has been effectively utilized to identify plaque burden in patients with coronary artery calcification (CAC). Calcium scoring is a score obtained from a computed tomography (CT) image using non-contrast imaging, which provides quantitative information regarding CAC and aids in assessing cardiovascular risk. A calcium score of zero Hounsfeild units can be obtained using CT calcium scoring which indicates no calcium is identified in the coronary arteries and is a strong negative risk predictor for coronary artery disease.

Early screening of SLE patients with CT calcium scoring could aid in early detection and treatment subsequently leading to delay of premature coronary atherosclerosis and future cardiac events in this patient population. Multiple studies have used calcium scoring as a method to measure arterial calcification in SLE patients. The Society of Cardiovascular Imaging has now endorsed the idea of obtaining a baseline calcium artery score with a repeat progression scan in 3-5 years. Calcium scoring has also been identified as an effective initial tool for stratification and identification of possible ACS. The various advantages of early calcium scoring signify the further research needed to fully understand and implement the advantages calcium scoring has to offer patients with SLE.

## Introduction and background

Systemic lupus erythematosus and cardiovascular disease risk

Systemic lupus erythematosus (SLE) and its unique presentations have perplexed physicians and have made treating the disease very complex. While the underlying mechanisms of SLE have yet to be fully elucidated, advances in understanding the etiology of SLE have led to potential mechanisms of disease pathophysiology. SLE pathogenesis has been characterized by the creation of cytoplasmic autoantibodies specific to self-nuclear and cytoplasmic antigens. Consequent immune complexes and free antibody production can lead to SLE disease pathology of various organs [1]. While SLE impacts multiple organ systems, the cardiovascular system appears to carry one of the highest disease burdens, especially the heart and coronary arteries [2].

**Figure 1:**
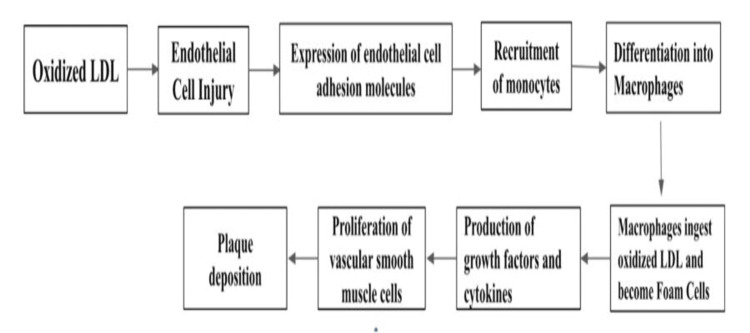
Flow diagram outlining the pathogenesis of atherosclerosis LDL: Low-density lipoprotein

One of the factors that likely contribute to the associated cardiovascular disease (CVD) burden in patients with SLE is the increased prevalence of atherosclerosis among these patients. Kiani and colleagues attributed this burden to the presence of higher levels of coronary calcification even when controlling for multiple traditional cardiovascular risk factors [3]. In addition, there appears to be upregulation of various cytokines in SLE patients that are associated with atherosclerosis [4]. Interferon alpha seems to be crucial for this process, as it is responsible for the upregulation of pro-inflammatory cytokines that include IL-6, IL-17, IL-21, IL-2, TNF-a, and IFN-gamma, resulting in excessive dendritic cell and consequent T-cell activation, ultimately leading to a loss of T-cell tolerance to self-antigens and the onset of autoimmune diseases [5,6]. In addition, interferon alpha plays a key role in the inhibition of endothelial progenitor cell production, resulting in diminished vascular repair and foam cell formation, leading to atherosclerotic build-up, and later calcification of the arteries [7]. Finally, leptin has been identified in elevated amounts in patients with SLE. Leptin at increased levels has been shown to lead to insulin resistance which makes it both atherogenic and pro-inflammatory. The elevated levels of leptin have furthered coronary inflammation and atherosclerosis in SLE patients [8]. The advanced coronary artery calcification (CAC) in these patients has been linked to early mortality in SLE patients [3,9]. Figure 1 displays the general pathogenesis of the deposition of atherosclerotic plaque [10,11].

Calcification and acute coronary syndrome

The progression and build-up of calcification within coronary arteries put patients, especially those with SLE, at a higher risk of developing acute coronary syndrome (ACS). ACS refers to conditions that result in decreased blood flow to the heart, commonly due to atherosclerotic plaque build-up, leading to ischemia and infarction [12]. ACS is a consequent effect of early atherosclerosis seen in SLE patients most likely due to the dysregulation of pro-inflammatory cytokine secretion [5-7]. Advanced atherosclerotic plaque development has been found to place patients with SLE at an elevated risk of developing a myocardial infarction [13,14]. In one study, Singh found that patients with SLE who developed ACS 5.5 years earlier, had a higher risk of developing an ST-elevation myocardial infarction and delayed recovery after myocardial infarction with diminished left ventricular function and left ventricular ejection fraction [15].

Calcification is an early risk identifier of atherosclerotic plaque burden and is a hallmark sign of advanced atherosclerosis [16]. The progression of atherosclerosis develops in various stages. First, injury to the endothelial cells provokes an inflammatory response and leads to cellular dysfunction. Chemokines attract circulating monocytes and T-lymphocytes to the site of injury. Both leukocytes and vascular smooth muscle cells (VSMCs) accumulate and grow within the intimal arterial wall. Macrophages that are activated as smooth muscle cells move from the media to the intima. The more permeable endothelium permits the entry of low-density lipoprotein (LDL) into the intima of the artery and macrophages can engulf these particles by phagocytosis. Lipid-laden macrophages are known as “foam cells'' and collections of these cells can create fatty streaks. In addition, activated macrophages secrete proinflammatory cytokines and induce VSMCs apoptosis. The elevated calcium as a result of apoptotic bodies causes the subsequent release of calcifying extracellular vesicles (cEVs) providing nucleation sites for initiating microcalcification in the plaque [17]. The microcalcification particles develop in four stages: cEV accumulation, aggregation, fusion of the cEV membrane, and mineralization [17]. In the mineralization stage, amorphous calcium phosphate transforms into mature crystalline hydroxyapatite particles [17]. With the progression of atherosclerotic disease, the microcalcifications combine and grow within the plaque’s core, fibrous cap, and medial tissue matrix. The evolution of atherosclerosis can form slow-growing, stable plaques not prone to rupture or more rapidly growing, unstable plaques with thin fibrin caps. Once a plaque ruptures, it can activate the clotting cascade and trigger an acute thrombosis.

Calcium scoring

Calcium scoring has been effectively utilized to identify plaque burden in patients with CAC [18]. The radiographic procedure results in a score from a computed tomography (CT) image using non-contrast imaging providing quantitative information regarding CAC and aids in assessing cardiovascular risk [19,20].

Currently, there are three scoring systems used to quantify the calcification in arteries, which include the Agatston score, volume score, and mass score [19]. Despite advances in CT scanning, the Agatston score remains the most widely used and is considered the standard score when referencing CAC [19]. Calcification is defined as a hyperattenuating lesion above 130 Hounsfield units (HU) with an area of three or more pixels. The Agatston score represents the total area of calcium deposits and is the sum of calcium in the right coronary, left anterior descending, and left circumflex arteries [21]. A score of zero indicates no calcium is identified in the heart and is a strong negative risk predictor for coronary artery disease (CAD), with a negative predictive value of 99%, sensitivity of 91%, and specificity of 64% [22]. A score of 101 to 400 HU indicates moderate plaque deposition. A score of greater than 400 is a sign of extensive disease. The higher the score, the greater the risk of heart attack. Calcium scoring was included in the multi-ethnic study of atherosclerosis (MESA) which is a risk assessment tool for 10-year coronary heart disease risk. The MESA risk protocol is the only risk assessment that considered coronary artery scoring for risk calculation, and since, has been internally and externally validated as an acceptable risk assessment measure [19]. Calcium scoring has been proposed as a tool to obtain a baseline measurement of calcification and to measure progression to better evaluate predictions of cardiovascular events, which may have a role in the management of patients with SLE [19].

Calcium scoring and SLE management

The risk of ACS is greatly elevated in patients with SLE. As a result, it is crucial to evaluate the early development and progression of atherosclerotic plaque in SLE patients using coronary artery calcium screening to prevent severe cardiovascular outcomes. Currently, it is understood that there are traditional risk factors associated with CAD including arterial hypertension, high cholesterol serum levels, obesity, and older age [23]. Meanwhile, these traditional risk factors do not entirely explain the pathogenesis of accelerated atherosclerosis in patients with SLE. While the mechanism of accelerated atherosclerosis in SLE is not fully understood, there have been a few studies investigating other nontraditional risk factors that may be contributing. These factors are summarized in Table 1.

**Table 1:** Significant findings and risk factors for coronary artery disease in SLE patients APS: Anti-phospholipid syndrome; BAFF: B-cell activating factor; CVD: Cardiovascular disease; LDL: Low-density lipoprotein; SLE: Systemic lupus erythematosus; VLDL: Very low-density lipoprotein

Source	Coronary Artery Disease Risk Factors in SLE Patients	Type of Study	Significant Findings	Limitations
Theodorou et al. [24]	BAFF	Experimental study	High BAFF was found to be significant in the association of CVD with SLE compared to the low-risk group.	The study does not evaluate the coronary arteries. Only the carotid and femoral arteries are evaluated for intimal media thickness.
Skamra and Ramsey-Goldman [25]	Corticosteroids	Literature review	This study stated longer corticosteroid use and higher dosages of corticosteroids were associated with increased CVD events.	The role of corticosteroid usage in SLE atherosclerosis is still unclear.
Jiménez et al. [26]	Antiphospholipid antibodies	Comparative study	This study found that SLE patients with secondary APS have a higher prevalence of plaques in the carotids.	The presence of preclinical atherosclerosis in SLE and primary APS has limited research conducted.
Svenungsson et al. [27]	Dyslipoproteinemia	Comparative study	One study compared SLE patients with CVD to those without and found dyslipoproteinemia was present significantly in SLE cases but not in SLE controls. It also found increased triglyceride concentrations in VLDL and LDL fractions.	Only decreased high-density lipoprotein levels have been established in SLE patients while LDL concentrations have been found to be similar to control patients.
Svenungsson et al. [27]	Homocysteine	Comparative study	This study saw a large discrimination in that the level of homocysteine was much higher in SLE patients with CVD versus those without.	Homocysteine pathogenesis in atherosclerosis is unclear.
Svenungsson et al. [27]	Lupus anticoagulant	Comparative study	The level of lupus anticoagulant was found to be much higher in SLE patients with CVD than without.	The study does not evaluate atherosclerosis in the coronary artery.
Svenungsson et al. [27]	anti-OxLDL, anti-MDA-LDL antibodies of IgG type	Comparative study	These antibodies were found to be significantly higher in patients with SLE and CVD than just SLE patients. Note these may just be markers of the disease and not causational risks.	The differences in the population studied may cause age bias in the results.

This literature review aims to investigate the proposed usage of CT calcium scoring in the primary prevention of ACS and future cardiac events in patients with SLE.

## Review

Methods

Article results were cultivated using the three databases PubMed, MEDLINE, and Clinical. All articles included in the results were limited to publication within the years 2000 to 2023. All articles were screened and reviewed with the chosen articles being both full-length articles and abstracts relating to calcium scoring and SLE included. Results were found using the search terms SLE, ACS, CT scan, and calcium scoring. All articles used focused on patients with SLE, calcium scoring, or ACS. All the articles also referred to or referenced an adult population. None of the studies used focused on the young child or adolescent population.

Results

Significant findings from the literature review are seen in Table 2.

**Table 2:** Results table displaying the significant findings supporting the usage of calcium scoring screening in SLE patients ACS: Acute coronary syndrome; CAC: Coronary artery calcification; CAD: Coronary artery disease; SLE: Systemic lupus erythematosus

Author	Title	Type of Study	Results/Significant Findings	Limitations of Study
Karrar et al. [28]	Coronary artery disease in systemic lupus erythematosus: a review of the literature	Literature review	- It was found that women aged between 35 and 44 years, had at least a 50-fold greater presence of CAD. - SLE was an independent risk factor for atherosclerosis.	- Does not directly address the usage of CT calcium scoring as a method of ACS prevention.
Katz et al. [29]	Systemic lupus erythematosus and increased prevalence of atherosclerotic cardiovascular disease in hospitalized patients	Experimental study	- Hospitalized male and female patients with SLE had higher rates of CAD and cardiovascular atherosclerosis as compared with matched patients without SLE. - Young women with SLE were at the greatest risk for atherosclerosis. SLE is an independent risk factor for atherosclerosis.	- Analysis was based on self-reporting and subject to reporting errors and bias. - Only hospital data sets were identified so possible underrepresentation of SLE patients.
Sayhi et al. [30]	Non-coronary cardiac manifestations of systemic lupus erythematosus in adults: a comparative study	Comparative study	- Patients with SLE present with an asymptomatic presentation during cardiac events.	- The study only assessed cardiac manifestations of SLE using echocardiography and not CT calcium scoring. - Does not address coronary artery manifestations in SLE patients.
Yiu et al. [31]	Pattern of arterial calcification in patients with systemic lupus erythematosus	Cross-sectional observational study	- The odds of patients with SLE having a calcium score above zero was 33.6 when compared to age-matched control groups.	- Detailed clinical assessment of disease severity of SLE was not performed. - Only CRP levels were taken to determine systemic inflammation.
Gartshteyn et al. [32]	Prevalence of coronary artery calcification in young patients with SLE of predominantly Hispanic and African-American descent	Comparative study	- Used calcium scoring as a measure to find coronary calcium burden. - Young patients as early as 21 years of age had a higher prevalence of CAC scores greater than zero. - Concluded that the data they collected regarding increased calcium scores in patients with SLE warrants early screening and cardio-protective measures.	- The participation rate was only 30% which allows for possible bias to be introduced.
Farshad et al. [33]	Utility of coronary calcium scoring (CCS) in connective tissue disorders (CTDs) for the evaluation of subclinical coronary atherosclerosis - a systematic review	Systematic review	- They evaluated the use of calcium scoring in connective tissue diseases such as SLE and found that patients with SLE experience significantly increased rates of CAC, which were identified with increased calcium scoring.	- Lack of statistical analysis to summarize study results and assist in eliminating bias.
Fernandez-Friera et al. [34]	Coronary CT and the coronary calcium score, the future of ED risk stratification?	Literature review	- They evaluated the diagnostic value of coronary artery calcium scoring in patients with possible ACS. - They found CT calcium scoring to be an effective initial tool for stratification and identification of possible ACS.	- The literature review only evaluates the usage of CT calcium scoring in an emergency department setting and not as a method of primary prevention.

Discussion

SLE is a chronic autoimmune disease with pathological implications affecting multiple organ systems, especially the cardiovascular system including the heart and coronary arteries. Patients with SLE are prone to premature atherosclerosis which commonly affects the coronary arteries. This commonly leads to patients with a predisposition for ACS and future cardiac events. The effects of SLE have become so impactful on the cardiac system that SLE is now an independent risk factor for premature atherosclerosis [13]. With the inflammatory and atherosclerotic effects of SLE presenting at an early age, early detection of atherosclerosis could aid in the early prevention of further atherosclerosis and limit future adverse cardiac events. Early screening of SLE patients with CT calcium scoring could present a possible solution for early detection of premature coronary atherosclerosis and aid in preventing future cardiac events in this patient population.

SLE Effects and Atherosclerosis

SLE is commonly known to have an effect on women of older age. In a literature review conducted by Karrar et al., the investigators identified that women aged between 35 and 44 years, had at least a 50-fold greater presence of CAD [28]. The investigators also found SLE to be a significant risk factor for coronary atherosclerosis, independent of classic risk factors. In another study by Katz et al., the researchers found that hospitalized male and female patients with SLE had higher rates of CAD and cardiovascular atherosclerosis as compared with matched patients without SLE [29]. They also found that SLE was an independent risk factor for atherosclerotic CVD and young women had the greatest risk as compared with matched control patients. Another concern is that patients with SLE have an asymptomatic presentation during cardiac events [30]. In a study conducted by Yiu et al., the author evaluated the prevalence and pattern of arterial calcification in patients with asymptomatic SLE. The authors found that the odds of patients with SLE having a calcium score above zero was 33.6 compared to age-matched control groups [31]. They also found that arterial calcification occurred early involving patients under the age of 40 and increased in prevalence as patients aged [31]. In all SLE age groups, they found that the coronary arteries were the most commonly calcified compared to other arteries in SLE patients [31]. With SLE patients having arterial calcification at such early ages as well as the asymptomatic presentation, this could present clinicians with a problem in the diagnosis and identification of ACS or early cardiovascular events. Clinicians could be less likely to identify cardiac manifestations in SLE patients due to the asymptomatic cardiac presentation and atherosclerosis occurring in younger individuals. Calcium artery scoring is currently recommended for asymptomatic cardiac imaging in individuals and could serve this population with its predictive value and its ability to classify patients as high-risk or low-risk. Whereas coronary angiography is only indicated in patients with current signs and symptoms of cardiac disease [35]. Finally, in a review conducted by Gartshteyn et al., the authors used calcium scoring in an effort to find out the coronary calcification burden in patients with SLE. Out of the 76 patients with SLE in the study, they found that 32 patients had coronary artery scores greater than zero. About 29% of the 18-32 years old stratified risk group showed increased coronary calcification. Patients as early as 21 years of age had a CAC score greater than zero. The authors further concluded that the data they collected regarding increased calcium scores in patients with SLE warrants early screening and cardio-protective measures [32]. The unique presentation and implications of SLE solidify the need for an imaging modality that can aid in the early detection of coronary atherosclerosis and reduce the risk of cardiovascular events. CT calcium scoring presents as a possible radiographic screening method for patients with SLE in an effort to find early coronary calcification and decrease future cardiac events. Early usage of statins before atherosclerosis has been proposed as a way of primary prevention for cardiac events in this patient population. In a study conducted by Schanberg et al., it was found that routine statin usage over three years had no significant effect on subclinical atherosclerosis progression in young SLE patients [36]. Many of the articles use calcium scoring as a method to evaluate arterial calcification in SLE patients and have found increased calcification which further justifies the usage of this modality as a screening method in efforts to decrease coronary calcification and prevent future adverse cardiac events.

CT Calcium Scoring Advantages

CT calcium scoring has gained traction in the primary prevention of CVD. Calcium scoring has been mainly used for its power of exclusion of those at risk. Patients who undergo calcium scoring and have a score of zero are at a low 10-year cardiovascular risk [37]. Studies using non-invasive CT calcium scoring have also shown that the greater the calcium score the higher the likelihood of patients developing future cardiac events [38]. In a systematic review done by Farshad et al., the authors evaluated the use of calcium scoring in connective tissue diseases such as SLE and found that patients with SLE experience significantly increased rates of CAC, which were identified with increased calcium scoring [33]. This study supports the use of calcium scoring in patients with SLE as a screening tool to determine the onset of coronary calcification and the risk of cardiovascular events. In addition, the Society of Cardiovascular Computed Tomography suggests obtaining a baseline calcium artery score and then repeating the scan as a progression scan in 3-5 years [19]. The basis for these guidelines is rooted in the idea that CAC progression can be followed with calcium scoring and could lead to treatment alteration and cardiac event prevention [19]. In a review conducted by Fernandez-Friera et al., the authors evaluated the diagnostic value of coronary artery calcium scoring in patients with possible ACS. After the conclusion of the review, they found CT calcium scoring to be an effective initial tool for stratification and identification of possible ACS. The authors concluded CT calcium scoring was an extremely valuable imaging modality due to its high negative predictive value and the additive diagnostic value the modality offers [34]. Aside from the imaging advantages CT calcium scoring offers, it has also been shown to be a cost-effective measure in the guidance of CVD treatment. In a study conducted by Valério et al., the authors found that coronary artery calcium scoring used to guide treatment decreased incremental costs and improved the quality of life in patients as compared with the use of conventional strategies [39]. The evidenced CAC identification and cost-effectiveness demonstrated by CT calcium scoring lend support to its use as a screening tool for SLE patients.

Limitations

Some limitations facing more usage of calcium scoring is the lack of research on the cost-effectiveness of the imaging as well as the effectiveness in aiding the risk assessment of individuals with CAC [19]. Further research could aid clinicians in their decision of whether to use early CT calcium scoring in SLE patients in efforts to identify early coronary atherosclerosis and prevent future cardiac events. Another limitation of coronary calcium scoring is that it cannot rule out the presence of noncalcified atherosclerotic plaques which have been shown to have greater instability and are more prone to rupture [21]. CT angiography could be a useful imaging modality to help in identifying the progression of noncalcified coronary plaque in SLE patients [40]. Furthermore, another consideration for improving CAC assessment is to further reduce radiation exposure. Studies have attempted to reduce radiation exposure, but these techniques have been associated with increased background noise and a lower CAC score than predicted [41]. Although there may be limited research on the usage of CT calcium scoring in patients with SLE, the evidence that has been collected and presented lends support to further exploring the implications of the usage of early CT calcium scoring in patients with SLE for the detection of early atherosclerosis and prevention of future cardiac events.

## Conclusions

CVD is the leading cause of death among patients with SLE and it appears that subclinical atherosclerosis develops early among individuals with SLE, which warrants early screening and protective interventions. One method commonly used as a non-invasive test for early detection of plaque build-up is CT calcium scoring. Recent studies have found that coronary artery calcium scoring has the potential to be a strong independent predictor of CVD. The build-up of calcification within coronary arteries places patients, especially those with SLE, at a higher risk of developing ACS. The early use of CT calcium scoring in patients with SLE could aid in the detection of early atherosclerosis and the prevention of future cardiac events. Meanwhile, CAC scores are limited in routine clinical practice due to radiation exposure and lack of widespread availability, despite the clinical value of CT calcium scoring. The coronary calcium scan may help patients with SLE understand their risk of heart disease and may possibly help guide clinicians in developing a treatment plan based on an individual's risk with the hope of halting the progression of CVD in SLE patients.
